# Aging in Cats: Owner Observations and Clinical Finding in 206 Mature Cats at Enrolment to the Cat Prospective Aging and Welfare Study

**DOI:** 10.3389/fvets.2022.859041

**Published:** 2022-04-04

**Authors:** Nathalie Dowgray, Gina Pinchbeck, Kelly Eyre, Vincent Biourge, Eithne Comerford, Alexander J. German

**Affiliations:** ^1^Institute of Life Course and Medical Sciences, University of Liverpool, Liverpool, United Kingdom; ^2^International Cat Care, Tisbury, United Kingdom; ^3^Institute of Infection, Veterinary and Ecological Sciences, University of Liverpool, Liverpool, United Kingdom; ^4^Royal Canin Research Centre, Aimargues, France; ^5^School of Veterinary Science, University of Liverpool, Liverpool, United Kingdom

**Keywords:** cat, feline, mature, middle-aged, survey, dental, welfare

## Abstract

Two hundred and six cats, aged between 7 and 10 years, from the North-west of the UK, were enrolled in a cat aging and welfare study to determine the frequency of age-related conditions and associations with husbandry, owner observations of physical appearance, activity and behavior. This is the largest study to date of mature cats that includes data from an owner questionnaire and clinical examinations. At enrolment, owners frequently reported physical changes (53%), behavioral changes (47%), changes to eating patterns (41%), and activity changes (40%) in their mature cats. On physical examination, 45% cats were in overweight condition and 12% were obese. A heart murmur was detected in 29% cats, whilst indirect systolic blood pressure (SBP) was >160 mmHg in 5% cats. Dental disease was present in 54% cats and was associated with a matted hair coat (*P* = 0.01), increased sleeping (*P* = 0.02), absence of gray hairs (*P* = 0.03), and increased irritability to other pets (*P* = 0.04). Abnormalities were evident in 58% of cats that allowed an orthopedic examination (OE) to be performed. These cats were older than cats with a normal OE (*P* = 0.01), and abnormal OE findings were associated with a matted coat (*P* = 0.03) and increased grooming (*P* = 0.04). Aazotaemia was present in 10% cats, and this was associated with cats being observed to “sniff their food and then walk away” (*P* = 0.04). Hyperthyroidism was diagnosed in 3% cats, who were older (*P* = 0.02), had a leaner BCS (*P* = 0.02) and lesser blood creatinine concentrations (*P* = 0.01). Hyperthyroid cats were also more likely to have increased liver enzyme activity and increased SBP (*P* = <0.001) compared with non-hyperthyroid cats. Of the 176 cats where all clinical assessments were conducted, only 12% had no evidence of any disease. Clinical abnormalities are commonly identified when thorough, clinical assessments are performed in mature pet cats visiting primary care practice.

## Introduction

In consensus guidelines for mature and senior cats, wellness examinations are recommended in cats over 10 years (y) of age (1–3) on an annual or 6-monthly basis. However, these recommendations are not reflected in what happens in general practice. In the UK, cats visit veterinarians only about half as often as dogs do (4) whilst, in the US, 37% of owners do not recall their veterinarian recommending annual examination and only 48% had taken their cat to the veterinary clinic in the preceding year (5). In a recent survey, 84% of UK cat owners were registered with a veterinarian, but only 64% were regularly vaccinated (6). Annual booster vaccinations are no longer recommended routinely and, instead, an approach tailored to the individual is preferred (7, 8); despite this, only 56% of practice owners have reported that they have a standardized approach which may lead to miscommunication if owners are seeing different staff members with practices (5). Whilst there is consensus agreement amongst feline veterinarians that mature (7–10 y) and senior (>10 y) cats should be examined every 6–12 months (1–3), there is less evidence on what age “senior” wellness appointments should be introduced, what health screening should occur, and what information should be gathered from owners to aid in early diagnosis of disease.

To date, most research into screening for age-related diseases has been undertaken in cats >9 y (9–12), with only two studies including cats aged 6–10 y (13, 14). In the first study, 56 cats aged 6–10 y were assessed alongside 44 cats aged >10 y (13); three (5%) of the cats aged 6–10 y had blood pressure measurements >160 mmHg, two (4%) were underweight, 24 (43%) were overweight and two (4%) had a heart murmur. On clinicopathological analysis, two (4%) of the 6–10y cats were hyperthyroid, six (11%) had a urine specific gravity of <1.035 and one (2%) had IRIS (international renal interest society) stage 2 chronic kidney disease (CKD) (13). In the second study, hematology, biochemistry, urinalysis and total thyroxine concentrations were assessed in 130 reportedly clinically-healthy cats aged 6–9 y who were attending veterinary practices for routine procedures (14). Findings indicating significant disease (e.g., anemia, CKD), or warranting further diagnostic investigations, were present in 25 (19%) cats (14).

Behavioral changes associated with aging in cats have been reported (15, 16), and such changes need to be recognized because they can signal the onset of chronic disease (e.g., hyperthyroidism, CKD) prompting investigations and treatment (15, 16). Given that such behavioral changes may develop before other systemic signs of illness, identifying them can facilitate early recognition of disease (17). In older cats, associations between behavioral changes and CKD, blindness, lower urinary tract infections, hyperthyroidism, osteoarthritis, heart disease, deafness, and dental disease have been reported (17). However, given that this study relied solely on owner-reported information on disease states rather than examinations undertaken by veterinary professionals, further research is required.

The aim of this study was to gather standardized information from both owners and veterinary assessments to determine the prevalence of disease, behavioral changes and changes in physical appearance in a large group of mature cats. We hypothesized that there would be associations between the diagnosis of diseases and the presence of owner observed changes and the cats' home environment and care.

## Materials and Methods

### Study Design and Ethical Approval

The Cat Prospective Aging and Welfare Study (CatPAWS) commenced in 2016, with the key aim of monitoring the health of pet cats during the aging process, facilitated by the establishment of the Feline Healthy Aging Clinic (FHAC) at the University of Liverpool, UK. Cats were enrolled at the FHAC between 7 and 10 years of age with the aim to monitor regularly thereafter using various clinical assessments (see below), data presented here are from their enrolment appointment only. Ethical approval for this study was granted by the University of Liverpool Veterinary Research Ethics committee (VREC491abcd) and the Royal Canin Ethical committee. At enrolment, all owners were provided with a detailed information sheet explaining the purpose of the study and gave written consent for their cat to be enrolled, which included permission to obtain their cat's medical records from its registered veterinary practice.

### Recruitment and Eligibility Criteria

Cats were recruited from three local veterinary practices in the Liverpool City region between February 2017 and October 2019. Recruitment was *via* direct invitation (e.g., letter or email) or advertising within the clinics, *via* social media, local, and national press and to University of Liverpool staff through institutional briefing emails. Cats were excluded if owners had requested not to be contacted by third party offers and marketing, their account was in debt at the registered veterinary practice, they were registered with a feline rehoming charity or if the cat was marked as a stray.

Eligible cats were those between 7 and 9 y, whose owners consented and were willing to visit the FHAC every 6 months. Cats were not excluded based on any pre-existing medical conditions, although some owners self-excluded their cats based on temperament. A required sample size of 385 cats was calculated using an online tool (www.epitools.ausvet.com.au) based on an estimated 50% prevalence of age-related disease in cats of this age group with 5% precision and 95% confidence intervals.

### Data Collection and Storage

#### Owner Questionnaire Data

Before attending their first appointment, owners completed the enrolment questionnaire (see Supplementary Material). In section one of the questionnaire, signalment data were captured including age, sex, neuter status, color, and breed. In section two, information about environment and lifestyle (including housing and physical resources) were recorded, whilst the third and fourth sections covered food and water resources, as well as eating and drinking behaviors. The fifth section covered their cat's health, previous and current illnesses, and any observed physical or activity changes from adult (3–6 y) to mature (7–10 y) life stages; the sixth section covered their cat's current behavior and any changes, whilst the seventh section covered details of the primary caregiver, the household and their relationship with the cat. In addition, medical records were obtained from the cat's registered veterinary practice.

#### Clinical Assessments

All clinical assessments undertaken were performed by the same veterinarian (ND). On arrival, respiratory rate was measured (RR1), the first cardiac auscultation was performed and the heart rate was recorded (HR1). Systolic blood pressure (SBP) was then measured using the Doppler method (Burtons BP Doppler or Thames medical CAT+ Doppler), and following consensus guidelines (18). All cats were weighed using portable electronic scales (V20 scale, Burtons, UK), and their body condition scored (BCS) using a 9-unit scale (19). A complete physical examination was then performed, which included a second respiratory rate measurement (RR2), second cardiac auscultation and heart rate measurement (HR2). Where cats were amenable, an orthopedic examination was performed involving a visual assessment of movement in the consultation room, musculoskeletal system palpation and manipulation of appendicular joints and skeletal spine, as previously described (20). Finally, cardiac auscultation was performed for a third time, and heart rate was once again measured (HR3).

Once these clinical assessments had been completed, a blood sample was collected for routine clinicopathological assessments. Phlebotomy was performed primarily from the jugular vein but, in cats exhibiting signs of discomfort during neck manipulation, the cephalic vein was used. Minimal restraint was used in most cases, with occasional towel restraint, and blood samples were not collected if the cat became excessively stressed. Blood was collected into tubes containing either EDTA, heparin or without anti-coagulant. Owners had already been supplied with a home collection kit for urine (Katkor, UK), to enable collection of a “free-catch” urine sample that could be brought to the practice on the day of their appointment. If this was not possible, an attempt was made to collect a sample by cystocentesis during the appointment, provided the cat was amenable and the bladder was of sufficient size.

#### Clinicopathological Assessments

Immediately after venepuncture, remaining blood in the syringe was used to measure glucose concentration using a glucometer validated for use in cats (AlphaTRAK, Zoetis, US). A complete hematological assessment, serum biochemistry panel, and total thyroxine (TT4) measurement were also performed. For cats seen between 2017 and most of 2018, samples were processed at the University of Liverpool Small Animal Teaching Hospital (SATH) laboratory. However, due to technical and staffing issues during the study period, in-house analysers were used to perform biochemistry panels (CatalystOne chemistry analyser, IDEXX laboratories) from November 2017 onwards, whilst in-house analysers were also used for hematological examinations (Procyte DX, IDEXX) and total thyroxine measurements (CatalystOne chemistry analyser, IDEXX laboratories) from July 2019 onwards. All urine samples were analyzed in-house either by the same registered veterinary nurse (Kelly Eyre) or veterinarian (ND). Urine specific gravity was measured using a refractometer, whilst biochemical tests were performed with urine dipsticks (Multistix 8 SG Reagent strips, Bayer) (21). For samples over 2 ml, 1 ml of urine was spun in a centrifuge for 5 min at 10 *g*, and the sediment was examined as both wet and dry samples.

#### Data Storage

A password-protected database management system (Microsoft Access, Microsoft Office, Version 2016, Microsoft Corp.) was used to store anonymised data, with cats being identified by a unique identification number. For data analysis, data were downloaded on to a computer spreadsheet (Excel, Version 2016, Microsoft Corp.). All data were stored in a manner that met requirements under the data protection act of 2018 (22).

### Diagnostic Criteria of Age-Related Disease

The following criteria were used to diagnose and determine disease states in the study population.

#### Azotaemia

A diagnosis of feline chronic kidney disease (CKD) usually requires evidence of sustained functional or structural kidney damage (23). Given that the data in this study were from a single time point, sustained damage could not be confirmed. Therefore, to be considered azotaemic and potentially at risk of having CKD, cats had to have a creatinine concentration >176.8 μmol/L, preferably in conjunction with a urine specific gravity (USG) <1.035 (9, 10).

#### Hypertension

For a diagnosis of hypertension, sequential measurements of SBP are usually required, showing persistent increases above reference limits (>160 mmHg), where there was no evidence of target organ damage (18). Given that, in the current study, SBP was only measured at a single time point, cats were classified as having hypertension when SBP was >160 mmHg or retinal examination revealed evidence of hypertensive retinopathy (18).

#### Hyperthyroidism

Cats were classified as having hyperthyroidism when total thyroxine concentration was greater than the reference interval (60 nmol/L) or when expected clinical signs were evident (e.g., weight loss, polyphagia), a total thyroxine concentration was >50 nmol/L and free thyroxine concentration was greater than reference interval (this was performed by the cats registered veterinary clinic and a number of external laboratories were used) (24).

#### Dental Disease

Dental disease was deemed to be present if the cat's gingivitis score was ≥2 (a score where clinical intervention (in the form of a dental procedure under general anesthesia; GA) would be advised) (25), feline odontoclastic resorptive lesions (FORL) were present, or there was evidence of stomatitis.

### Statistical Analysis

Statistical analysis was performed using an online statistical environment (R version 4.0.0) (26). Additional packages “sp” (27) and “Rcpp” (28) were used to examine and describe the data. All reported *P-*values are two-sided, and the level of assumed statistical significance was *P* ≤ 0.05.

Descriptive statistics were used to summarize the results from the questionnaire data, with frequencies reported using numbers and percentages.

Continuous datasets were first assessed for normality using histograms and the Shapiro-Wilk test; methods of statistical assessment and the reporting of summary data depended on the distribution of the dataset in question. In this respect, normally-distributed data were compared with either the paired Student's *t-*test or the 2-sample Student's *t-*test), or one-way ANOVA (with Tukey *post-hoc* comparisons), whilst results are expressed as mean, standard deviation, minimum and maximum. Data not following a normal distribution were assessed with the Wilcoxon signed-rank test (paired data), the Mann-Whitney test or the Kruskal-Wallis test (with Dunn's test used for *post-hoc* comparisons), whilst results are expressed as median and interquartile ranges (IQR). Further, repeated-measures ANOVA was used to assess the multiple heart rate measurements (Tukey test used for *post-hoc* comparisons). Relationships were explored using either Pearson's correlation (normally-distributed data) or Spearman's rank correlation (data that were not normally distributed). Prevalence and 95% CI were calculated using an online tool (https://epitools.ausvet.com.au/).

Relationships between all owner-observed changes reported in the questionnaire and findings on clinical investigations for azotaemia, hyperthyroidism, musculoskeletal disease and dental disease were made using either the Chi-squared test or Fisher's exact test (where small values were present). Weighted Cohen's kappa analysis was performed to assess the agreement between owner assessment of their cat's body condition from the questionnaire data and veterinary-assessed BCS at the enrolment visit. Logistic regression analysis was performed to assess the association between hyperglobulinaemia and the presence of clinical anomalies.

## Results

### Part One: Questionnaire

#### Demographics and Husbandry

Two hundred and six cats were enrolled between February 2017 and October 2019. The median age of cats enrolled was 8 y (IQR 7–9 y), with most (178, 86%) being of mixed breeding (domestic shorthair or longhair), whilst pedigree breeds were represented in the remaining 28 (14%) of cats (Supplementary Table 1). One hundred and nine cats were male (53%), 107 (98%) of which were neutered and 97 (47%) were female, 94 of which were neutered (three female cats were of unknown neuter status) (Supplementary Table 1).

One hundred and forty-seven owners enrolled cats into the study, with 32 (22%) of them enrolling more than one cat; most owners were female (119, 81%) and aged between 25 and 54 y (106, 72%). One hundred and nine (74%) had previously owned a cat. Not unexpectedly, given that this was a University veterinary practice, many owners (72, 49%) had postgraduate or professional qualifications; under half (60, 41%) were in full-time work, and most (98, 67%) lived in either a single person household or as part of a couple. When asked about their relationship with the cat, 103 (70%) considered them to be a family member over a pet, pest controller or best friend (Supplementary Table 2). Forty-seven cats (23%) were indoor only; for 12 cats, this was because there was no outside space available, whilst the remaining 35 cats were not allowed outdoors. The remaining 158 cats (77%) went outside, with 81 (51%) of these only being allowed outside when the owners were home. Litter trays were used exclusively by 107 (52%) of the cats, with a further 56 (27%) toileting in both litter trays and outdoors and 42 (21%) toileting outside only (Supplementary Table 3). In 183/197 (93%) cats, a proprietary dry food was fed either exclusively or as part of the daily ration, whilst 163/197 (83%) cats were given proprietary wet food (either in pouches or tins). A variety of other foods were also fed, accounting for up to 55% of intake in some cats (Supplementary Table 4), whilst treats were given to 100/197 (51%) of the cats, and 21/200 (11%) were given a dietary supplement.

#### Owner-Reported Heath and Preventive Healthcare

Annual vaccinations had been given to 92/205 cats (45%) (Supplementary Table 5), whilst 180/205 cats (88%) and 155/202 (77%) cats were treated for external and internal parasites, respectively (Supplementary Table 6). One hundred and eight of 203 (53%) cats were reported to have had one or more episodes of ill-health before enrolment, 88 (44%) reported no previous episodes of ill-health and 7 (4%) could not remember. The most common owner-recalled episodes of ill health were gastro-intestinal disease 26 (13%), skin disease 19 (9%), and lower urinary tract disease 17 (8%) (Supplementary Table 7).

Owners reported that their veterinarian had advised dental treatment in 48/198 cats (24%). Of these, dental work under general anesthesia (GA) was recommended for 34 cats (71%) cats, with a further three cats (6%) recommended both dental work under GA and either diet change or tooth brushing. Tooth brushing alone was recommended for 7/48 cats (14%), whilst a combination of tooth brushing and dietary changes was recommended for 2/48 (4%) cats. Dental work under GA had been performed prior to study enrolment in 29/37 (78%) cats, with 25/29 (86%) requiring dental extractions.

Based on the colloquial categories in the enrolment questionnaire, 5/199 owners (3%) considered their cat to be underweight, 22/199 (11%) lean, 95/199 (48%) ideal, 57/199 (28.5%) overweight, and 20/ 199 (10%) obese. Owners reported that the body condition of their cat was stable in 103/177 cats (58%), had increased in 45/177 (25%) cats and had decreased in 19/177 (11%) cats, whilst the remaining owners (11/177; 6%) did not know. Owners that considered their cat to be overweight were more likely to report that their cat had gained weight as they had aged (*P* < 0.001).

Vomiting in the previous year was reported in 130/205 (63%) cats, and a further 17/205 (8%) reported observing vomitus but were unsure of its source in a multi-cat household. Of the cats reported to have vomited, 70/130 (54%) vomited less than once a month, 34/130 (26%) a few times a month, 13/130 (10%) weekly and 4/130 (3%) several times a week, whilst no frequency was reported in the remaining 9/130 (7%) cats. Hairballs were always present in the vomitus in 22/130 cats (17%), occasionally present in 78/130 cats (60%) and never present in 24/130 cats (18%), whilst owners of the remaining 6/130 cats (5%) did not know. There was no association between the presence of hairballs and the frequency of vomiting (*P* = 0.33).

#### Owner-Observed Changes Up to Middle Age

Physical changes were commonly-reported (109/206, 53%; **Table 4** and Supplementary Table 8), with halitosis and gray hairs (23, 11%, respectively) being the two most common. Activity changes were less commonly reported (83/206, 40%) but, of those reported, increased sleeping (52, 25%) and reduced outside activity (38, 18%) were the most common activity changes observed. Changes to eating pattern were reported in 85 cats (41%), whilst behavior changes were reported in 96 cats (47%). “Sniffing food and then walking away” was the most common change in eating pattern reported (37, 18%), whilst an increased demand for attention (56, 27%) along with an increased display of affection toward people (55, 27%) were the most common behavior changes reported.

#### Previous Health History

Of the cats with a record of vaccination, 84/104 (79%) were vaccinated against feline leukemia and 7/104 (7%) were vaccinated against rabies. Of the 143 cats where previous medical records were available (Supplementary Table 9), 53 (37%) owners reported in the questionnaire that their cat had no previous episodes of ill health despite there being evidence of this in the cats' medical records. Conditions most likely not to be reported in the questionnaire were trauma (6/53, 11%), upper respiratory tract infection, feline lower urinary tract disease (5/53, 9%), and skin disease (5/53, 9%).

### Part Two: Clinical Examination Findings

#### Physical and Physiological Parameters

A complete clinical assessment was possible in 176/206 cats (85%). Abnormalities in the orthopedic examination and dental disease were the most common findings, followed by heart murmurs (Table 1). There was no association between age (*P* = 0.10) or BCS (*P* = 0.10) and the number of morbidities determined in those cats. A summary of data on weight, BCS and physiological parameters is presented in Table 2. Three cats (1%) were underweight (BCS <4/9), 86 (42%) were in ideal weight (BCS 4–5), 93 (45%) were overweight (BCS 6–7), and 24 (12%) had obesity (BCS 8–9). Respiratory rate was measured twice during the consultation in 197 cats, with the second reading (RR2) being greater than the first (RR1; *P* = 0.01). Heart rate was measured on three occasions during the consultation in 175 cats, with the first (HR1) being greater than both the second (HR2, *P* < 0.001) and third (HR3, *P* < 0.001), but there was no difference between the second and third (*P* = 0.66). On cardiac auscultation, a heart murmur was detected in 59/206 cats [29%, 95% confidence interval (95%-CI) 23–35%] at one or more of the cardiac examinations during their appointment. Results from all three time-points were available from 169 euthyroid cats, and a heart murmur was present in 50 (30%, 95%-CI 23–37%) of these cats. Of these 169 cats, 32 (64%) had a murmur at HR1, 27 (54%) at HR2, and 30 (60%) at HR3. The detection of a heart murmur varied within individual cats; a heart murmur was present on all three occasions in 14 cats (28%), on two occasions in 13 cats (26%) and on a single occasion in 23 cats (46%). Heart murmurs were grade I-II/VI in 41 cats (82%) and grade III-VI/VI in nine cats (18%), with the latter being more likely to be present on all three occasions (*P* < 0.001). There was no significant difference in heart rate between the cats with and without a heart murmur at each time point (HR1 *P* = 0.65, HR2 *P* = 0.24, HR3 *P* = 0.53), and heart rate was not associated with grade of murmur (*P* = 0.54). Systolic blood pressure (SBP) results are summarized in Table 2. SBP was increased (>160 mmHg) in 9/194 cats (5%, 95%-CI 2–9%), although this was thought to be due to the stress of the examination in two of these cats. In a further two cats (1%), SBP was 150–160 mmHg, but changes consistent with hypertension were also present on retinal examination, and both cats were diagnosed with CKD on their biochemistry.

**Table 1:** A summary morbidities found in 176 cats which completed a full clinical examination.

	**None**	**OE**	**DD**	**HM**	**AZO**	**SBP**	**HT**
Total	22 (12%)	104 (59%)	95 (54%)	55 (31%)	19 (11%)	8 (4%)	6 (3%)
OE	—	31 (30%)	57 (60%)	33 (60%)	11 (58%)	6 (75%)	4 (67%)
DD	—	57 (55%)	19(20%)	34 (62%)	12 (63%)	5 (62%)	5 (8%)
HM	—	33 (32%)	34 (36%)	8 (4%)	7 (37%)	6 (75%)	6 (100%)
AZO	—	11 (11%)	12 (13%)	7 (13%)	2 (10%)	2 (25%)	0 (0%)
BP	—	6 (6%)	5 (5%)	6 (11%)	2 (10%)	1 (12%)	3 (50%)
HT	—	4 (4%)	5 (5%)	6 (11%)	0 (0%)	3 (38%)	0 (0%)

*All data are the number (percentage) of clinical abnormalities found. Gray shaded boxes indicate the number of cats where a single condition was determined. The donominator for calculating the percentage is the total number of cases for that condition, for example, 57 cats had abnormalities on their OE and dental disease, 55% of cats with OE abnormalities had dental disease but 60% of cats with dental disease had OE abnormalities. OE, abnormalities on orthopedic examination; DD, dental disease; HM, heart murmur; AZO, azotaemia; SBP, Systolic blood pressure >160 mmHg and cats with hypertensive retinopathy; HT, hyperthyroidism*.

**Table 2:** Summary of weight, BCS, and physiological parameters during clinical assessment conducted in 206 cats aged 7–10 y.

**Variable**	**Measurements**
Weight (kg)	4.6 (4.0–5.3)
Body condition score (1–9)	6 (5–7)
Respiratory rate 1 (*n* = 206)	52 (41–62)
Respiratory rate 2 (*n* = 197)	54 (42–68)
Systolic blood pressure (mmHg)	128 (114–139)
Heart rate 1 (*n* = 206)	187 (22.8)
Heart rate 2 (*n =* 191)	178 (25.6)
Heart rate 3 (*n* = 183)	180 (22.8)

*All results are presented as median (interquartile range), except for heart rate which is reported as mean (standard deviation)*.

#### Dental and Oral Examination

Dental disease was present in 111/205 cats that allowed an oral examination (54%, 95%-CI 47–61%), with findings including FORL, periodontal disease, severe gingivitis, and often a combination of all three. Feline odontoclastic lesions were present in 59/203 cats (29% 95%-CI 23–36%), whilst stomatitis was present in 8/187 cats (4%, 95%-CI 2–8%).

#### Orthopedic Examination

Full details of the OE performed in 184 of the cats are available in the Supplementary Tables 10, 11; of these, 182/206 (88%) cats allowed most of the examination to be completed. Abnormalities, indicating possible musculoskeletal disease, were evident in 107 cats (59%, 95%-CI 52–66%), with these cats being older than those whose OE revealed no abnormalities (*P* = 0.01), but with no difference in the BCS between groups (*P* = 0.82).

#### Hematology and Serum Biochemistry

Complete details of hematological and serum biochemical measurements are available in the Supplementary Tables 12, 13. Hematology measurements were available from 172/206 (83%) cats. There was no association between the cats' age and either red blood cell count (*R* = −0.018, *P* = 0.81), haematocrit (*R* = −0.084, *P* = 0.27), or white blood cell count (*R* = −0.071, *P* = 0.35). Leucocytosis (six cats, 4%, 95% CI 2–7%), or a decreased haematocrit (3, 2%, 95%CI 0.6–5.0) were uncommon and, therefore, no comparisons were made with the presence of other diseases.

Serum or plasma biochemistry measurements were obtained from 189/206 cats (92%), with the temperament of the remaining 17 cats (8%) not allowing venepuncture. Electrolyte concentrations were available from 176 (85%) cats where a sufficient volume of blood was collected to enable measurement. Glucose concentration was greater than renal threshold (~12 mmol/L) in six cats (3%), but none were diagnosed with diabetes mellitus because they did not exhibit persistent hyperglycaemia or typical clinical signs (29). The blood glucose concentrations of one enrolled diabetic cat, which was currently on insulin therapy, were within the reference interval. Hypercalcaemia was present in one cat, whilst hypercholesterolaemia was present in 57 cats (30%). Alanine aminotransferase (ALT), alkaline phosphatase (ALP) and total bilirubin were assessed together: in six cats (3%), there was mildly increased ALT activity, whilst two cats (1%) had mildly increased ALP and ALT activity. In one further cat (1%), both ALP and ALT activity were moderately increased, whilst in one further cat (1%), there were concurrent increases in both ALP and ALT activity and bilirubin concentration.

In 23 cats (12%), creatinine concentration was above the reference range; in 10 of these cats, urinalysis was available and urine specific gravity (USG) was <1.035 in three of these cats. Nineteen (10%, 95%-CI 7–15%) of the cats were azotaemic, as defined in the methods section. There were no significant differences in weight or BCS between the cats with or without azotaemia (Table 3). Thirty cats (16%) had hyperglobulinaemia, and cats with the dental disease and azotaemia had increased odds of being the presence of hyperglobulinaemic [odds ratio 4.7 (1.8–14.9 95% CI) and 3.5 (1.4–9.3 95% CI), respectively].

**Table 3:** Relationship between weight, BCS, and azotaemia in 189 cats at enrolment.

	**Azotaemic**	**Non-azotaemic**	***P*-value**
	**(*n* = 19)**	**(*n* = 170)**	
Weight (kg)	4.8 (3.70–5.36)	4.6 (3.97–5.31)	0.96
BCS (1–9)	5 (5–6)	6 (5–7)	0.226

*Results presented as median and IQR*.

#### Total Thyroxine Concentration

Total thyroxine concentration was measured in 182 cats (88%), with no results being less than reference interval, and only 6 (3%, 95%-CI 2–7%) being above reference interval, suggestive of hyperthyroidism (see Supplementary Table 13). Compared with euthyroid cats, hyperthyroid cats were older (Median age 9.4 vs 8 y, *P* = 0.02) and leaner (median BCS 4 vs. 6, *P* = 0.02) but there was no difference in weight (median weight 4.05 vs. 4.65 kg, *P* = 0.60). Cats with hyperthyroidism also had a faster respiratory rate at both time points during their physical examination, compared with euthyroid cats (RR1 78 vs. 53, *P* = 0.02; RR2 88 vs. 58, *P* = 0.02). However, there was no difference in heart rate at any time point (HR1 208 vs. 186, *P* = 0.11; HR2 193 vs. 178, *P* = 0.21; HR3 180 vs. 182, *P* = 0.76). Further, unlike in euthyroid cats, a sequential decrease in heart rate across the three time points was not evident in hyperthyroid cats (*P* = 0.20). Heart murmurs were present in all 6 hyperthyroid cats, and they also had greater blood pressure compared to euthyroid cats (155 vs. 127 mmHg *P* = 0.01). Increased ALP and ALT activity was seen in 2/6 and 4/6 hyperthyroid cats, respectively.

#### Urinalysis

Urinalysis was performed in 41/206 cats (20%) at their enrolment visit. Median USG was 1.050 (IQR 1.042–1.050). Only five cats had a USG of < 1.035 suggestive of inappropriately concentrated urine (23).

### Part Three: Associations Between Husbandry, Owner Observations, Medical Records, and Clinical Examination Findings

#### Outdoor Access

There was no association between cats with outdoor access and either vaccination status (*P* = 0.48) or previous episodes of ill health, but cats with outdoor access were more likely to receive both regular ectoparasite (*P* < 0.001) and endoparasite treatment (*P* = 0.02). There was no difference in BCS at enrolment between indoor cats and cats with outdoor access (median BCS 6 for both groups, *P* = 0.37). Further, from prior medical records, there was no relationship between cats with outdoor access and a medical record of traumatic injury (*n* = 15, *P* = 0.07), fracture (*n* = 5, *P* = 0.32), cat bite abscess (*n* = 13, *P* = 0.11), upper respiratory tract infection (*n* = 33, *P* = 0.19), lower urinary tract disease (*n* = 28, *P* = 0.82), or dermatitis (*n* = 22, *P* = 0.08).

#### Diet

Owners of cats whose daily food intake comprised ≥50% proprietary dry food were more likely to observe them drinking (*P* = 0.01), but there was no relationship between the proportion of the diet comprising proprietary dry food and the presence of dental disease at enrolment (*P* = 0.87). There was also no relation between BCS at enrolment and the frequency of feeding (*P* = 0.34) or the proportion of proprietary dry food in the diet (*P* = 0.39).

#### Owner

There was no association between previous cat ownership and the use of external parasite treatments, internal parasite treatments or vaccination status. Educational qualifications of owners were also not associated with use of external parasite treatment or internal parasite treatment, but there was a significant association between educational qualifications and vaccination status (*P* < 0.001) with a greater proportion of owners with post-graduate or professional qualifications stating that their cats received annual vaccinations.

### Disease Status and Observations

Multiple disease states were determined in individual cats (Table 1). Twenty-two (12%) of cats were healthy, 61 (35%) were diagnosed with a single condition, 67 (38%) with two conditions, 16 (95) with three, 6 (3%) with four, and 4 (2%) with five different conditions.

#### Azotaemia

Azotaemia was associated with an increased frequency of owners reporting their cat “*sniffing at food then walking away*” (*P* = 0.04; **Table 5**), but no association with frequency of owner-observed drinking (*P* = 0.08) or in the owners' response to the question “*have you noticed you cat drinking more as they have got older?*” (*P* = 1.00). There was also no difference between azotaemic and non-azotaemic cats in terms of whether or not they had been observed vomiting in the previous year (*n* = 120, *P* = 0.41).

#### Hyperthyroidism

Cats with hyperthyroidism at enrolment were more often observed drinking frequently or very frequently compared with euthyroid cats (6/6, *P* = 0.03), but there was no difference in either reported changes to drinking behavior over time (*P* = 0.137) or increased drinking (*P* = 0.08). The owners of 5/6 cats with hyperthyroidism observed vomiting, but there was no relationship between the observation of vomiting (*P* = 0.66) or the frequency of vomiting (*P* = 0.22) and hyperthyroidism.

#### Dental Disease

Dental disease at enrolment was associated with the presence of a matted coat (*P* = 0.01), increased sleeping (*P* = 0.02) and a lack of gray hairs developing (*P* = 0.03) but not halitosis, mastication on one side of the mouth or “messier” eating and dropping food (Table 4). However, a previous history of dental disease was associated with owner-reported changes in eating behavior (*P* = 0.01), mastication on one side of the mouth (*P* = 0.02), “messier” eating (*P* = 0.03), and dropping food (*P* = 0.02). Whilst halitosis as their cat aged was associated with a previous recommendation of dental treatment (*P* = 0.01).

**Table 4:** Significance of owner observations and associations with health status on enrolment in 206 cats aged 7–10 y.

**Group**	**Observation^a^**	**AZO^b^**	**HT^b^**	**DD^b^**	**OE^b^**
Physical changes	Halitosis (23, 11%)	0.48	0.54	0.14	0.83
	Gray hair (23, 11%)	0.44	1.00	**0.03**	1.00
	Dullness of coat (18, 9%)	0.40	0.45	0.33	0.31
	Matted coat (14, 7%)	0.33	0.13	**0.004**	**0.03**
	Skeletal points more prominent (12, 6%)	1.00	1.00	0.77	1.00
Activity changes	Increased sleeping (52, 25%)	0.41	0.15	**0.015**	0.49
	Reduced outside activity (38, 18%)	0.12	1.00	0.11	0.57
	Increased appetite (25, 12%)	0.44	0.51	0.53	0.37
	Sleeping in different places (16, 8%)	1.00	0.35	0.18	0.78
	Reduced jumping (15, 7%)	0.62	0.35	0.60	0.56
	Increased grooming (14, 7%)	1.00	1.00	0.27	**0.04**
	Increased drinking (12, 6%)	1.00	0.08	0.08	0.35
Changes to eating patterns	Sniffing food then walking away (37, 18%)	**0.04**	1.00	0.86	1.00
	Increased eating and demanding food (29, 14%)	0.28	0.57	0.69	0.66
	Increased food left in bowl (29, 14%)	0.28	0.18	1.00	0.52
	Demanding food then not eating it (26, 13%)	0.30	1.00	0.83	0.27
	“Messier” eating (24, 12%)	0.70	1.00	0.83	0.17
	Reduced consumption of kibble (22, 11%)	0.06	1.00	0.82	0.11
	Dropping food (20, 10%)	1.00	1.00	0.43	0.90
	Reduced speed of eating (15, 7%)	0.62	1.00	0.18	0.15
	Mastication on one side of mouth (11, 5%)	0.30	0.33	0.07	1.00
Behavior changes	Increased demand for attention (56, 27%)	0.60	1.00	1.00	0.46
	Increased affection toward people (55, 27%)	1.00	1.00	0.82	0.50
	Increased vocalization at night (26, 13%)	1.00	1.00	1.0	0.69
	Increased vocalization both day and night (17, 8%)	0.63	1.00	1.00	0.82
	Increased irritability toward people (18, 9%)	0.62	1.00	0.62	0.24
	Increased irritability toward other pets (13, 6%)	1.00	0.06	**0.04**	0.08
	Increased nocturnal wakening (9, 4%)	0.18	1.00	0.51	1.00

a *The numbers in brackets represent the number and percentage of cats with that observation*.

b *Results represent 2-sided P-values from either Chi-squared test or Fishers exact test (conducted when group numbers were small), with those reaching the threshold for statistical significance (P < 0.05) highlighted in bold*.

*AZO, azotaemia; HT, hyperthyroidism; DD, dental disease; OE, abnormalities on orthopedic examination*.

#### Abnormal Orthopedic Examination Findings

A matted coat (*P* = 0.03) and increased grooming behavior (*P* = 0.04) were associated with abnormal findings on OE (see Table 4).

#### Abnormal Body Condition

Both overweight and obesity were more common (overweight 31/45, 69%; obesity 9/45, 20%) in cats whose owners reported that their cat had gained weight as they aged (45/166) than those reporting either no change (102/166, 61%) or that their cat had lost weight (19/166, *P* < 0.001). There was fair agreement between owner assessments of body condition and enrolment BCS (weighted kappa 0.41, 95%-CI 0.33–0.49, *P* < 0.001); however, a proportion of owners of cats in overweight condition believed that their cat was in ideal body condition and a proportion of owners with cats in appropriate body condition considered their cat to be underweight (Table 5).

**Table 5:** Comparison of body condition score and owner assessment of body condition.

	**BCS 3/9**	**BCS 4–5/9**	**BCS ≥6/9**
	**(*n* = 2)**	**(*n* = 81)**	**(*n* = 115)**
**Owner assessment**			
Underweight	0	7 (9%)	70 (61%)
Normal	0	54 (66%)	41 (36%)
Overweight	2 (100%)	21 (25%)	4 (3%)

*BCS, Body condition score; n, number of cats*.

## Discussion

In the current study, detailed clinical assessments were performed to determine the health of mature (7–10 y) cats. The demographics of the cohort were broadly similar to those reported in other UK based population studies of cats outside of the narrow age range selected (4, 30–32). Clinical abnormalities suggestive of chronic disease were present in most (154/176) cats that allowed a complete clinical assessment to be performed. Given that chronic diseases were commonly identified in these cats, practices should consider performing such clinical assessments regularly, for example as part of a wellness plan. Such a strategy could facilitate early diagnosis, with the potential for improving outcomes. One challenge with such an approach is the cost of the procedures, which might dissuade some owners from participating (33). However, the fact that only 12% of this population were considered to be completely healthy suggests that such an approach may be valuable both from a cost-benefit and welfare point of view. Orthopedic abnormalities and dental disease were particularly common in this cohort, both of which could affect patient wellbeing. Multiple comorbidities were common as indicated in reviews and guidelines of aging cats (3, 15, 34), although previously published studies on prevalence of multiple comorbidities are lacking. In canine studies, mean morbidity score (number of disease states present at that time point) increases with age across breeds but, to date, there are no similar studies in cats (35).

The apparent prevalence of orthopedic examination (OE) abnormalities was 59% in cats of this cohort. Although data from primary care practices in the UK suggest a 2% prevalence of degenerative joint disease (DJD) (30), this condition is known to be under-recognized in cats (36). Retrospective and cross-sectional studies analyzing feline radiographs for changes consistent with feline OA and DJD suggest a prevalence of 22–92% depending on the population examined and the methods used (37–41). Given that OE alone is poorly sensitive and specific for detecting radiographic DJD and OA (20, 41), the fact that radiographs were not performed, the apparent prevalence may be an overestimate or an underestimate of the true prevalence, as OE alone may increases the number of both false positive and false negative responses. The presence of a matted hair coat was associated with abnormal findings on OE, possibly associated with effects of musculoskeletal disease on grooming behavior, as was also highlighted in previous research (41, 42).

Dental disease was present in over half of the current cohort of cats at enrolment, including FORL, periodontal disease and marked gingivitis, and this might explain why dental treatment had already been recommended for 24% of the cats before study enrolment. In another study, the prevalence of dental disease and periodontal disease was 15 and 14%, respectively, in cats of all ages (30). The prevalence of gingivitis increases from 0 to 6 y (25), whilst severity of periodontal disease is also associated with age (43). Periodontal disease was present in 96% of cats in a research colony (mean age 6 y), with 13% having aggressive periodontitis (44). This is significantly greater than the prevalence reported in our study, likely due to the methodology for diagnosis of dental disease, whereby diagnosis was made with radiology under general anesthesia rather than visual assessment alone. The fact that dental therapy had already been performed in 29 cats before study enrolment, might also have contributed to the lower prevalence in the current study.

Dental disease at enrolment was associated with owners reporting a matted coat and that their cat was sleeping more. These owner observations might suggest that cats have oral pain, given that the behavioral signs of chronic pain in cats included an absence of grooming, reluctance to move, withdrawal or hiding, decreased activity, avoidance of brightly-lit areas and closing of eyes (45). There was an association between the presence of dental disease and the cat being less likely to have developed gray hairs, but the reason for this is not known and could be due to the small sample size or an inflated type I error due to multiple hypothesis testing on the same data. To control for this excluding associations that do not have a biological basis could be considered. Further, although no association was found between the type of food and the presence of dental disease at enrolment, the findings are limited by the fact that we did not consider prior dietary changes as a result of previous dental treatment or advice. Associations were found with cats previously advised to have dental treatment and changes to their eating behaviors. The majority of these cats had required a dental procedure under GA, implying that these eating behavior changes, the owners had observed, were due to pain from previous dental disease or due to dental extractions. However, no such association was found between eating behaviors and the cats with dental disease present at enrolment. Therefore, it is likely that the dental disease in cats requiring previous intervention was more severe, and it was this that caused the changes to eating behavior.

Heart murmurs were common in the cats of this cohort, with the apparent prevalence being 29%, which is higher than the prevalence reported in a previous study (5%) (30), but less than that found by Payne et al. (up to 41%) (46). Given that both these studies were based in the UK, the difference is likely to be due to the study methodology. The first was a retrospective study and reviewed electronic patient records from primary care veterinary practices, whilst the second was a prospective cohort of healthy cats age > 6 months from rehoming centers, where cardiac auscultation was performed on up to three occasions, resulting in 1,630 diagnostic auscultations from 780 cats (46). The lesser prevalence of heart murmurs found in our cohort compared to this study could either reflect a decreased prevalence in this population, or be due to the environment in which the cats were examined. Whilst the cardiac auscultation was performed up to three times during the appointment, the environment was a first opinion veterinary practice based next to a busy road. This might have had made it difficult for an optimal cardiac auscultation to be performed.

The prevalence of azotaemia in this cohort was 10%. In cats, a diagnosis of CKD requires evidence of sustained functional or structural kidney damage of at least 3 months' duration (23). As data were collected from a single time point, not all azotaemic cats necessarily had CKD. It was also not always possible to collect a contemporaneous urine sample for measurement of specific gravity and, therefore, true prevalence of CKD in this cohort is not known. The overall population prevalence of CKD in the UK is estimated to be 1% (47), with prevalence increasing with age in cats over 8–10 y (48). Further, in the USA, prevalence of up to 41% is reported in cats aged 5–10 y, 42% in cats aged 10–15 y, and 81% in cats aged 15–20y (49). This study included IRIS (international renal interest society) stage I CKD cases and is likely to contain more false positives than comparable studies that only include later stages of CKD. Annual incidence of CKD in previously healthy geriatric cats is between 18 and 36% within a year (9, 10). The difference between the two studies was explained by a slight difference in the median age, 15 y (9) compared to 13 y (10), of the cohorts at baseline. Therefore, it is expected that the prevalence of CKD will increase as the cats in the CatPAWS cohort get older. Weight loss in cats with CKD on average starts to occur 3 years prior to its diagnosis (50), and small changes in the cats eating behavior early in the disease process may be playing a role in this weight loss. Associated with the presence of azotaemia, were owner reports of the cats “sniffing at food then walking away.” Early recognition of this change of consumption would enable tailoring of the calorie intake to reduce or prevent the weight loss associated with CKD and may alert to the early presence of disease. However, it is worth noting that that number of cats with azotaemia was small in this cohort and further investigation of this finding is required.

The prevalence of hyperthyroidism in this cohort was 3%, which was the same as the estimate from a previous UK study (30), albeit increasing to 9% in cats ≥ 10 y (51) and 6% in cats >9 y (11). Other studies, conducted in Hong Kong (51) and Japan (52), have reported the prevalence of hyperthyroidism to be 4 and 9%, respectively. In a further prospective cohort study assessing the development of hyperthyroidism in a population of cats >9 y, the annual incidence was 7% (11). It is expected that the prevalence of hyperthyroidism will also increase in cats of the current cohort as they age. The relationship with hyperthyroidism and increased liver enzyme activity, hypertension and the presence of a heart murmur were not unexpected, since they are findings known to be associated with hyperthyroidism (24).

Based on a measurement of SBP >160 mmHg, the apparent prevalence of hypertension in the current study cohort was 5%. Cats with systolic blood pressure (SBP) of 160–180 mmHg are considered to have a moderate risk of target organ damage, with those having SBP >180 mmHg being at high risk (18). However, to diagnose hypertension, SBP measurements >160 mmHg without evidence of target organ damage (e.g., retinal changes) on at least two separate occasions are required (18). Given that SBP was only measured once in the current study, prevalence might have been over-estimated. That said, retinal examination was performed in most cats and changes consistent with hypertensive retinopathy (e.g., retinal oedema and hemorrhage) were found in two of them (both with SBP 150–160 mmHg and concurrent azotaemia). Furthermore, the measurements were taken in the clinic, rather than at home, and differences between SBP measurements taken at home and in the clinic environment can differ by as much as 31 mmHg (52), thus leading to an overestimation of hypertension prevalence. Previous studies in pet cats have shown that hypertension is more common in cats aged ≥10 y (53, 54) and in cats with CKD (55, 56) or hyperthyroidism (55, 57). In this cohort, SBP >160 mmHg was present in 3/6 cats with hyperthyroidism. Age, sex, neuter status, demeanor and a history of being a stray are all associated with increased SBP (58); but were not assessed in this study due to the narrow age range and high proportion of neutered cats in the cohort. Since SBP increases as cats age (59), the prevalence of hypertension in this cohort is likely to increase in the future.

The combined prevalence of overweight and obese body condition in this cohort was 57%, whilst only 2% were in underweight condition. In a previous study where a 5-unit BCS scale was used, 12% of cats were in overweight or obese condition (BCS 4-5/5), and middle-age (around 7 y) was a risk factor whilst only 2% of cats were in underweight (BCS 1/5) condition (60). In other UK studies, the prevalence of overweight and obesity was 39%, in cats from the Glasgow region (61), and 7% from a study of electronic patient records (30), with this latter result likely being an under-estimate, given the reliance on veterinary professionals formally recording body condition, a method associated with significant under-reporting in companion animals (61, 62). Bodyweight in cats increases up to 9 y of age and then tends to decrease thereafter (63), with the age range of 7–11 y being a risk factor for obesity in Australian cats (64). Therefore, the high prevalence of overweight and obesity in the current cohort of cats might partly be explained by the fact that the age of enrolment coincided with the period of peak risk.

There was fair agreement (weighted kappa 0.41) between owner and veterinarian assessments of body condition in the current study, which is consistent with previously published research (65). However, 34 and 39% of cats of normal or overweight BCS were misclassified by their owner (Table 5), with owners of cats in normal body condition being more likely to classify them as being underweight and overweight cats being more likely to be classified as normal. This is consistent with the literature showing cat owners are more likely to underestimate their cats body condition (66).

There was no relationship between BCS on enrolment and the proportion of cats fed proprietary dry food, which contrasts with a previous study (67). Given the different ages of cat included in these two studies (1 vs. 7–10 y), a possible explanation would be that any impact of dry food on weight gain diminishes with time. However, since both studies were observational, causality cannot be confirmed and further studies are required.

In this study, 39% of owners reported an episode of vomiting at least once a month; and 77% of owners reported always or sometimes seeing hairballs in the vomitus. Few other studies are available for comparison: in a survey of 48 cats from a UK feline-only clinic, 27% of cats had reportedly vomited at least one hairball in the last year (68), suggesting that hairballs might be less prevalent than the current study results suggest. Nevertheless, given that between a quarter and three quarters of pet cats vomit hairballs, more research is needed into causes and consequences. No relationship was found between observed vomiting and the presence of either azotaemia or hyperthyroidism. Given that associations between vomiting and both CKD and hyperthyroidism have previously been reported (23, 24), the lack of association in the current study might instead reflect the small numbers of hyperthyroid and CKD cats in this cohort or that they may be in an earlier stage of the disease.

Episodes of trauma, skin disease, urinary tract disease and upper respiratory tract disease were all more commonly recorded in medical history but not captured in owner recollections. This might be because owners do not associate such signs with “ill-health,” not least given that they tended to be short transient episodes. Nonetheless, it highlights the importance of including both owner and veterinary information in future studies of health and wellness in pet cats.

Most cats in this cohort were indoor but also had outdoor access (77%), which is less than reported in an earlier UK study where 87% of cats had similar access (69). This difference might be explained by the fact that the veterinary clinics enrolling cats were located in a central city region. Cats with outdoor access were more likely to receive regular endo- and ectoparasite treatment, likely reflecting an owner opinion that indoor-only cats are of less risk of parasitism. Although cats living indoors are reported to be a greater risk of obesity (67, 70), no association was seen between housing and BCS in the current study.

Unsurprisingly, cats whose diet comprised fed ≥ 50% dry food were more likely to be observed drinking by their owner. Therefore, type of diet needs to be considered before concluding that polydipsia is present when an owner observes their cat drinking. There was also no association between azotaemia and drinking frequency, and owners of azotaemic cats were more likely to report that they did not know if their cat had been drinking more as they aged. This suggests that polydipsia might be an insensitive clinical sign, at least in the early stages of CKD development. Conversely, cats with hyperthyroidism were often observed to be drinking frequently or very frequently, suggesting that this might be a more important clinical sign for this endocrinopathy.

About half of the cats in the current study were either unvaccinated or their vaccinations had lapsed., which is similar to a 2019 UK survey where 41% of cats did not have regular boosters (71) and a 2009 survey where 60% of cats (median age 7 y) had been vaccinated in the previous year (31). Cats whose owners had either a postgraduate or professional qualification were more likely to be current with their vaccinations, suggesting a possible education effect, however this may also reflect a bias in the owner population of this cohort as many were recruited from the universities veterinary practice. In contrast, 88% of cats were treated for ectoparasites and 77% for endoparasites, which are broadly similar results to those of a previous study (72).

Owner-observed changes were frequent in this cohort, with physical, activity or behavioral changes noted in 47, 60, and 53% of cats, respectively. The most common activity changes reported in the current study were increased sleeping (25%) and decreased outdoor activity (18%). Owners generally considered changes in behavior in cats around middle age to be positive, such as demanding more attention (27%) and becoming more affectionate toward people (27%). Increased sociability with people has also been previously reported in 36–52% of senior and geriatric cats (17).

Cat owners in our cohort were predominantly female (81%), which is consistent with a previous study reporting cat ownership in the north west of the UK (73). Female owner responders are also more likely to report cat ownership than male responders in pet ownership profiles (74). Most owners in the cohort were aged between 25 and 54 y (71%), consistent with other studies which have reported less cat ownership in the over 65 s (73, 74). Single-person households and couples were the most common (67%) cat owners, which is noteworthy given that cat ownership has previously been associated with families (73). The age of the cats recruited may explain this difference, as families may be more likely to have kittens and or young cats. Seventy-one percent of owners in the current study were either graduates, post-graduates, professionals or had vocational training; these findings are consistent with other studies whereby positive associations between education level or professional occupation cat ownership have been reported (73, 74).

The study has several limitations that should be acknowledged. First, data were only collected from a single point in time from this cohort and, therefore, cannot provide information about progression of disease. Related to this, examination at a single time point makes it difficult to diagnose some diseases (e.g., CKD and hypertension) with confidence. Additionally, associations only could be determined between owner reported cat behaviors and clinical findings, no causal link could be established. To address this, the cohort will continue to be monitored during the aging process, to enable such longitudinal data to be gathered, following cats that were disease free at enrolment to determine the timeframe between behavioral changes and the occurrence of disease. A second limitation was the fact that 206 cats were recruited, which was less than the 385 cats that the sample size calculation suggested and for some disease numbers were small meaning our prevalence estimates should be interpreted with caution and the power to detect associations was low. Furthermore, these cats belonged to only 147 owners and there is likely to be clustering of some management practices within owners. Finally, diagnostic imaging was not performed on cats with abnormalities on OE or a heart murmur and, as a result their relationship to underlying disease processes could not be determined.

In conclusion, detailed clinical assessments were performed in a population of mature cats (7–10 y) attending primary care practices, and clinical abnormalities were commonly identified. Such detailed assessments could form the basis of a wellness plan for cats as they age. Further, some owner-observed physical and behavioral changes (e.g., matted hair, increased sleeping, irritability, and sniffing food then walking away) were associated with the presence of chronic disease, and knowledge of these could facilitate earlier diagnosis, through raising owner awareness and encouraging them to seek veterinary assessment more promptly, with the potential for improving outcome.

## Data Availability Statement

Outside of the original contributions presented in the study included in the article and Supplementary Material, further inquiries can be directed to the corresponding authors.

## Ethics Statement

The animal study was reviewed and approved by University of Liverpool Veterinary Research Ethics Committee and the Royal Canin Ethical Committee. Written informed consent was obtained from the owners for the participation of their animals in this study.

## Author Contributions

ND, GP, EC, AG, and VB: conceptualization, review, and editing. ND: data collection, curation, statistical analysis, and drafting of the original manuscript. KE: data collection and curation. All authors contributed to the article and approved the submitted version.

## Funding

This study was funded by Royal Canin.

## Conflict of Interest

At the time the study was performed, ND was undertaking a post-graduate studentship funded by Royal Canin, a division of Mars Petcare. Since October 2020, ND has been employed by International Cat Care, but also holds a part-time post-doctoral research position at the University of Liverpool, funded by Royal Canin. AG and KE are employees of the University of Liverpool whose positions are funded by Royal Canin. AG has also received financial remuneration and gifts for providing educational material, speaking at conferences, and consultancy work. VB is an employee of Royal Canin. The remaining authors declare that the research was conducted in the absence of any commercial or financial relationships that could be construed as a potential conflict of interest.

## Publisher's Note

All claims expressed in this article are solely those of the authors and do not necessarily represent those of their affiliated organizations, or those of the publisher, the editors and the reviewers. Any product that may be evaluated in this article, or claim that may be made by its manufacturer, is not guaranteed or endorsed by the publisher.
