# Supplementary material for: Aging in Cats: Owner Observations and Clinical Finding in 206 Mature Cats at Enrolment to the Cat Prospective Aging and Welfare Study
Source: Front Vet Sci. 2022 Apr 4;9:859041. doi: 10.3389/fvets.2022.859041 (PMC9014291; doi:10.3389/fvets.2022.859041)
Supplement: Supplementary file 1 [file Data_Sheet_2.docx]

**Supplementary Material**

**Table 1**: Demographics for 206 middle-aged UK cats enrolled between February 2017 and October 2019

| **Age** | **Age (Years)** | 8 (IQR 7-9) |
| --- | --- | --- |
|  | *Age known* | 95 (46%) |
|  | *Age estimated* | 102 (50%) |
|  | *Not answered* | 9 (4%) |
| **Sex** | *Male* | 109(53%) |
|  | *Female* | 97 (47%) |
| **Breed** | *Domestic short hair* | 132 (64%) |
|  | *Domestic medium hair* | 24 (12%) |
|  | *Domestic long hair* | 22 (11%) |
|  | *Pedigree* | 28 (14%) |
|  | *Ragdoll*  *Persian*  *British short hair*  *Siamese*  *Bengal*  *Oriental*  *American exotic*  *Ocicat, Balinese, Sphynx, Maine Coon, Egyptian Mau, Somali, Russian Blue* | 4  4  3  3  3  2  2  1 respectively |
| **Neuter status** | *Neutered* | 201 (98%) |
|  | *Entire* | 2 (1%) |
|  | *Unknown* | 3 (1%) |
| **Coat colour** | *Black or black & white* | 83 (40%) |
|  | *Tabby or tabby & white* | 39 (19%) |
|  | *Ginger or ginger & white* | 31 (15%) |
|  | *Tortoiseshell* | 21 (10%) |
|  | *Grey or grey tabby* | 18 (9%) |
|  | *Other* | 14 (7%) |
| **Cat acquisition** | *Friend or relative* | 77 (37%) |
|  | *Rescue* | 53 (26%) |
|  | *Stray* | 34 (16%) |
|  | *Breeder* | 15 (7%) |
|  | *Born at home* | 6 (3%) |
|  | *Pet shop* | 3 (1.5%) |
|  | *Other* | 15 (7%) |
|  | *Not answered* | 3 (1.5%) |
| **Age at acquisition** | *Kitten <3mo* | 60 (29%) |
|  | *Kitten ≥3-6mo* | 37 (18%) |
|  | *Adolescent (˃6-12mo)* | 21 (10%) |
|  | *Young adult (˃12mo-2Y)* | 27 (13%) |
|  | *Adult (˃2Y-5Y)* | 36 (17.5%) |
|  | *Older cat (˃5Y-9Y)* | 14 (7%) |
|  | *“adult”* | 3 (1.5%) |
|  | *Not answered* | 8 (4%) |
| **Number of cats in house** | *Only cat* | 72 (35%) |
|  | *One other cat* | 68 (33%) |
|  | *Two or more cats* | 61 (30%) |
|  | *Not answered* | 5 (2%) |

Y = year, mo = month, IQR = interquartile range, results are expressed as absolute number and percentage.

**Table 2**: Owner demographics for 147 owners with 206 cats who enrolled between February 2017 and October 2019

| *Cats enrolled per owner* | *No. cats* | No. owners |
| --- | --- | --- |
|  | 1 | 115 (78%) |
|  | 2 | 24 (16%) |
|  | 3 | 5 (3%) |
|  | 4 | 1 (0.7%) |
|  | 5 | 1 (0.7%) |
|  | 14 | 1 (0.7%) |
| *Gender of owner* | Female | 119 (81%) |
|  | Male | 25 (17%) |
|  | Not answered | 3 (2%) |
| *Age range (years)* | Under 24 | 2 (1%) |
|  | 25-34 | 40 (27%) |
|  | 35-44 | 30 (20%) |
|  | 45-54 | 36 (24%) |
|  | 55-64 | 21 (14%) |
|  | 65 + | 18 (12%) |
| *Previously owner cats* | Yes | 109 (74%) |
|  | Other people in the house have | 11 (7.5%) |
|  | First cat | 26 (18%) |
| *Education level* | No additional | 9 (6%) |
|  | GCSE / O-level | 16 (11%) |
|  | A level | 11 (8%) |
|  | Undergraduate / vocation | 33 (22%) |
|  | Postgraduate / profession | 72 (49%) |
|  | Not answered | 6 (4%) |
| *Current employment* | Full-time work | 60 (41%) |
|  | Part time work | 20 (14%) |
|  | Retired | 29 (20%) |
|  | Not employed | 15 (10%) |
|  | Student | 7 (5%) |
|  | Other | 14 (9%) |
| *Household* | Couple | 54 (37%) |
|  | Single | 44 (30%) |
|  | Young family (children <10Y) | 23 (16%) |
|  | Older family (children 11-18Y) | 9 (6%) |
|  | Adult family (children >18Y) | 12 (8%) |
|  | Shared house | 3 (2%) |
| *Time lived at current house* | >1 year | 9 (6%) |
|  | >1 year & < 3 years | 30 (20%) |
|  | >3 years & <5 years | 21 (14%) |
|  | > 5 years | 84 (57%) |
| *Smoking in household* | No | 98 (66%) |
|  | Yes: in last 7 years but not currently | 24 (16%) |
|  | Yes: currently | 22 (15%) |
| *Relationship with cat* | Best Friend | 41 (28%) |
|  | Family member | 103 (70%) |
|  | Pet | 31 (21%) |
|  | Pest controller | 2 (1%) |
|  | Other | 7 (5%) |

No = Number, results are expressed as absolute number and percentage.

**Table 3**: Summary of environmental husbandry for 206 middle-aged cats enrolled between February 2017 and October 2019

|  |  | Number (%) of cats |
| --- | --- | --- |
| **House type** | *Terraced house* | 83 (40%) |
|  | *Semi-detached* | 59 (29%) |
|  | *Apartment/flat* | 47 (23%) |
|  | *Detached house* | 15 (7%) |
|  | *Not answered* | 2 (1%) |
| **Number of bedrooms** | **Number of cats** | **Access to whole house** |
| *1* | 18 (9%) | 18 (100%) |
| *2* | 74 (36%) | 69 (93%) |
| *3* | 76 (37%) | 65 (86%) |
| *4* | 26 (13%) | 15 (57%) |
| *5 or more* | 10 (5%) | 5 (50% ) |
| **Outdoor access** | *Indoor only* | 47 (23%) |
|  | *Garden* | 91 (44%) |
|  | *Yard* | 56 (27%) |
|  | *Terrace* | 8 (4%) |
| **Toileting** | *Litter tray only* | 107 (52%) |
|  | *Outside only* | 42 (21%) |
|  | *Litter tray and outside* | 56 (27%) |
|  | *Other* | 1 (1%) |
| **Are people at home** | *Always* | 82 (40%) |
| **during the day?** | *Variable hours away* | 57 (28%) |
|  | *Regular hours away* | 31 (15%) |
|  | *No* | 35 (17%) |
| **Sleeping places** | *Owner’s bed(s)* | 161 (78%) |
|  | *Couches* | 152 (74%) |
|  | *Cat beds* | 111 (54%) |
|  | *On top of furniture* | 97 (47%) |
|  | *Floor* | 91 (44%) |
|  | *People’s laps* | 68 (33%) |
|  | *Window still* | 64 (31%) |
|  | *By radiator* | 62 (30%) |
|  | *Under furniture* | 54 (26%) |
| **Enrichment** | *Scratching post* | 162 (80%) |
|  | *Cat toys* | 152 (75%) |
|  | *Cat bed* | 146 (72%) |
|  | *Climbing frame* | 73 (36%) |
|  | *Feline pheromones* | 41 (20%) |
|  | *No enrichment* | 7 (3.5%) |

Results are expressed as absolute number and percentage.

**Table 4**: Summary of diets from 197 cats’ enrolled between February 2017 and October 2019: Dietary types being fed and the proportion of their total diet this dietary type accounts for.

| *Type Diet* | *Number of cats given* | *Proportion of total diet* |
| --- | --- | --- |
| *Commercial dry (kibble)* | 183 (93%) | 2-100% |
| *Pouch or tinned* | 163 (83%) | 2-100% |
| *Prepared raw food diet* | 4 (2%) | 5-25% |
| *Tinned fish* | 30 (15%) | 1-25% |
| *Fresh fish* | 14 (7%) | 1-10% |
| *Cooked chicken* | 43 (22%) | 1-20% |
| *Raw chicken* | 6 (3%) | 1-55% |
| *Other raw meat* | 6 (3%) | 1-30% |
| *Other cooked meat* | 18 (9%) | 1-20% |
| *Dairy products* | 14 (7%) | 0.5-2% |
| *Cat treats* | 100 (51%) | 0-30% |
| *Other* | 16 (8%) | 5-25% |

Results are expressed as absolute number and percentage and the range of percentages expressed when owners were asked what proportion of the cats’ diet this accounted for.

**Table 5**: Vaccination status prior to enrolment for 205 middle-aged cats enrolled between February 2017 and October 2019

| Annual vaccinations | 92 (45%) |
| --- | --- |
| Adult vaccinations but not up to date | 43 (21%) |
| Kitten vaccinations | 33 (16%) |
| Not vaccinated | 25 (12%) |
| Don’t know | 12 (6%) |

Results are expressed as absolute number and percentage.

**Table 6**: Routine parasiticide use prior to enrolment for 206 middle-aged cats enrolled between February 2017 and October 2019

| ***External parasite treatment reported for 205 cats*** | | |
| --- | --- | --- |
| *Treated* | Treated | 180 (88%) |
|  | Not treated | 25 (12%) |
| *Type of treatment used* | Spot-on | 168 (82%) |
|  | Spray | 4 (2%) |
|  | Tablet | 3 (1%) |
|  | Other | 3 (1%) |
|  | Not answered | 26 (13%) |
| *Where was treatment* | Vets | 95 (46%) |
| *purchased* | Pet store | 52 (25%) |
|  | Supermarket | 19 (9%) |
|  | Online | 9 (4%) |
|  | Not answered | 30 (15%) |
| *How frequently is* | As required | 79 (39%) |
| *treatment given* | Monthly | 58 (28%) |
|  | In summer only | 18 (9%) |
|  | Other | 25 (12%) |
|  | Not answered | 25 (12%) |
| ***Internal parasite treatment reported for 202 cats*** | | |
| *Treated* | Treated | 155 (77%) |
|  | Not treated | 47 (23%) |
| *Where was treatment* | Veterinary practice | 52 (26%) |
| *purchased* | Pet store | 44 (22%) |
|  | Supermarket | 9 (4%) |
|  | On-line | 3 (1%) |
|  | Not answered | 54 (27%) |
| *How frequently is* | As required | 45 (22%) |
| *treatment given* | Quarterly | 39 (19%) |
|  | Biannually | 45 (22%) |
|  | Annually | 15 (7%) |
|  | Other | 11 (5%) |
|  | Not answered | 47 (23%) |

Quarterly = 3-monthly, Biannually = 6-monthly, results are expressed as absolute number and percentage.

**Table 7**: Owner recalled episodes of ill health in 203 cats aged 7-10 years enrolled between February 2017 and October 2019.

| **Body system affected** | **Number of cats** |
| --- | --- |
| *Gastrointestinal* | 26 (13%) |
| *Skin* | 19 (9%) |
| *Lower urinary tract* | 17 (8%) |
| *Upper respiratory tract / ocular* | 13 (6%) |
| *Trauma* | 13 (6%) |
| *Dental / oral* | 6 (3%) |
| *Fight/abscess* | 5 (2%) |
| *Non-specific illness* | 5 (2%) |
| *Musculoskeletal* | 3 (1%) |
| *Asthma* | 3 (1%) |
| *Kidney* | 3 (1%) |
| *Uterine* | 2 (1%) |

Results are expressed as absolute number and percentage.

**Table 8**: Owner-observed changes up to middle-age reported in 206 cats aged between 7-10 years enrolled between February 2017 and October 2019.

|  | *Not answered* | *No change* | *Common changes reported* |
| --- | --- | --- | --- |
| *Physical changes* | 11 (5%) | 109 (53%) | Halitosis (23, 11%)  Grey hair (23, 11%)  Dullness of coat (18, 9%)  Matted coat (14, 7%)  Skeletal points more prominent (12, 6%) |
| *Activity changes* | 11(5%) | 83 (40%) | Increased sleeping (52, 25%)  Reduced outside activity (38, 18%)  Increased appetite (25, 12%)  Sleeping in different places (16, 8%)  Reduced jumping (15, 7%)  Increased grooming (14, 7%)  Increased drinking (13, 6%) |
| *Changes to eating pattern* | 17 (8%) | 85 (41%) | Sniffing food then walking away (37, 18%)  Increased eating and demanding food (29, 14%)  Increased food left in bowl (29, 14%)  Demanding food then not eating it (26, 13%)  ‘Messier’ eating (24, 12%)  Reduced consumption of kibble (22, 11%)  Dropping food (20, 10%)  Reduced speed of eating (15, 7%)  Mastication on one side of mouth (11, 5%) |
| *Behavioural changes* | 14 (7%) | 96 (47%) | Increased demand for attention (56, 27%)  Increased affection towards people (55, 27%)  Increased vocalisation- night (26, 13%)  Increased vocalisation - day and night (17, 8%)  Increased irritability towards people (18, 9%)  Increased irritability towards other pets (13, 6%)  Increased nocturnal wakening (9, 4%) |

Results are expressed as absolute number and percentage.

**Table 9:** Summary of medical records and health conditions diagnosed by body system available from 143 middle-aged cats enrolled between February 2017 and October 2019

| **Record available by cat** | *Complete medical record* | 125 (61%) |
| --- | --- | --- |
|  | *Partial medical record* | 12 (6%) |
|  | *Minimal medical records* | 6 (3%) |
|  | *No medical records* | 63 (30%) |
| **Vaccination Status** | *Vaccinated* | 104 (50%) |
|  | *Not vaccinated* | 48 (23%) |
|  | *No medical record of vaccination* | 54 (26%) |
| **Summary of health conditions** | **Body system affected** |  |
|  | *Gastro-Intestinal* | 4 (3%) |
|  | *Skin* | 22 (15%) |
|  | *Lower urinary tract* | 28 (19%) |
|  | *Upper respiratory tract / ocular* | 34 (24%) |
|  | *Trauma* | 15 (10%) |
|  | *Dental / oral* | 24 (17%) |
|  | *Fight / abscess* | 13 (9%) |
|  | *Lump removal* | 3 (2%) |
|  | *Musculoskeletal* | 8 (6%) |
|  | *Asthma* | 2 (1%) |
|  | *Kidney* | 1 (1%) |
|  | *Heart disease* | 2 (1%) |

Results are expressed as absolute number and percentage.

**Table 10**: Summary of subjective visual observation of movement in 184 cats who completed an orthopaedic examination (OE) at enrolment between February 2017 and October 2019.

|  | *Number of cats recorded* | *Yes* | *No* |
| --- | --- | --- | --- |
| *Limping* | 179 | 3 (2%) | 175 (95%) |
| *Moving easily* | 176 | 156 (85%) | 20 (11%) |
| *Willing to jump / climb* | 179 | 103 (56%) | 76 (41%) |

Results are expressed as absolute number and percentage.

**Table 11**: Results from the physical orthopaedic examination (OE) of 184 mature cats at enrolment between February 2017 and October 2019

|  | ***Left forelimb*** | ***Right forelimb*** |
| --- | --- | --- |
| *Carpus Thickening* | 181 (98.5%) normal; 2 (1%) not examined, 1 (0.5%) could not manipulate | 179 (97.5%) normal; 5(2.5%) not examined |
| *Carpal ROM* | 180 (97.5%) normal, 1 reduced ROM, 1 could not manipulate, 2 not examined | 178 (97%) normal, 2 not examined, 1 could not manipulate |
| *Carpal pain* | 179 (97.5%) no pain, 3 not assessed, 2 low grade pain | 179 (97%) no pain, 1 marked pain, 4 not assessed |
| *Elbow thickening* | 166 (90%) normal, 15 (8%) mild/moderate thickening, 1 severe thickening, 1 not assessed, 1 could not manipulate. | 167 (91%) normal,15 (8%) mild/moderate thickening, 2 not assessed |
| *Elbow ROM* | 149 (81%) normal, 24 (13%) reduction in ROM, 2 not assessed, 9 (5%) could not manipulate. | 150 (81.5%) normal, 28 (15%) reduction in ROM, 1 not assessed, 6 cannot manipulate |
| *Elbow pain* | 160 (87%) no pain, 2 (1%) moderate pain, 18 (10%) mild pain, 4 (2%) not assessed, | 158 (86%) no pain, 20 (11%) mild pain, 6 not assessed, |
| *Shoulder thickening* | 180 (98%) Normal, 2 mild/moderate thickening, 1 could not manipulate, 1 not assessed | 179 (97.5%) normal, 3 mild/moderate thickening, 2 not assessed |
| *Shoulder ROM* | 146 (79%) normal, 28 (15%) reduced ROM, 9 (5%) could not manipulate, 1 not assessed | 144 (78%) normal, 33 (18%) reduced ROM, 6 could not manipulate, 1 not assessed |
| *Shoulder pain* | 156 (85%) no pain, 24 (13%) mild pain, 4 not assessed | 154 (84%) no pain, 24 (13%) mild pain, 6 not assessed |
|  | ***Left Hindlimb*** | ***Right Hindlimb*** |
| *Tarsal thickening* | 178 (97%) normal, 6 not assessed | 176 (96%) normal, 8 not assessed |
| *Tarsal ROM* | 172 (93.5%) normal, 6 could not manipulate, 6 not assessed | 92% Normal, 4.5% could not manipulate, 3.5% not assessed |
| *Tarsal pain* | 174 (94%) no pain, 10 not assessed | 173 (94%) no pain, 11 not assessed |
| *Stifle thickening* | 169 (92%) normal, 9 (5%) mild/moderate thickening, 6 not assessed | 91% Normal, 5% mild to moderate, 4% not assessed |
| *Stifle ROM* | 134 (73%) normal,16 (9%) reduced ROM, 28 (15%) could not manipulate, 6 not assessed | 136 (74%) normal, 8% reduced ROM, 28 (15%) could not manipulate, 6 not assessed |
| *Stifle pain* | 162 (88 %) no pain, 8 (4%) mild pain, 1 moderate pain, 13 not assessed | 162 (88%) no pain, 8 (4%) mild pain, 14 not assessed |
| *Patella luxation* | 171 (93%) no, 2 yes, 11 not assessed | 166 (90%) no, 3 yes, 15 not assessed |
| *Coxofemoral joint ROM* | 66 (36%) normal, 70 (38%) reduction in ROM, 44 (24%) no extension possible, 4 not assessed | |
| *Coxofemoral joint Pain* | 114 (62%) no pain, 33 (18%) mild pain, 6 (3%) moderate pain, 31 not assessed | |
| ROM = Range of motion, mild pain = mild withdrawal or resistance to manipulation, moderate pain = moderate withdrawal / body tenses / may orient to site examined / may vocalise or increase vocalisation during manipulation, marked pain = turns towards site, forcible withdrawal from manipulation, vocalise, hiss or bite during manipulation. | | |

**Table 12**: Summary of electrolyte results from 176 middle-aged cats enrolled study between February 2017 and October 2019.

|  | *Mean* | *Min* | *Max* | *SD* |
| --- | --- | --- | --- | --- |
| *Sodium (mmol/L)* | 157.5 | 146.0 | 164.0 | 3.15 |
| *Potassium (mmol/L)* | 3.94 | 3 | 5.7 | 0.38 |
| *Chloride (mmol/L)* | 120.1 | 100.0 | 131.0 | 4.01 |
| Min = minimum values, max=maximum value, SD = standard deviation. | | | | |

**Table 13**: Summary of serum or plasma thyroxine (TT4) results from 182 middle-aged cats enrolled between February 2017 and October 2019.

|  | *TT4(nmol/L)* | *Age (y)* | *Weight (kg)* | *BCS* |
| --- | --- | --- | --- | --- |
| *Euthyroid (176 )* | 26 (21-31) | 8 (7-9) | 4.6 (3.97-5.35) | 6 (5-7) |
| *Hyperthyroid (1 early)* | 67.8 | 11 | 7.8 | 7 |
| *Hyperthyroid (5 )* | 158 (136-174) | 9.4 (8-10.28) | 4.1 (2.86-4.93) | 4 (3-5) |
| Results are expressed as median and interquartile range, BCS = Body condition score | | | | |

**Table 14**: Summary of serum biochemistry results obtained from 189 middle-aged cats enrolled between February 2017 and October 2019

|  | *Mean* | *Median* | *Min* | *Q1* | *Q3* | *Max* | *SD* | *Cats with measurements outside the reference ranges* |
| --- | --- | --- | --- | --- | --- | --- | --- | --- |
| *Glucose (mmol/L)* | 6.5 | **6.0** | 3.4 | **5.2** | **7.0** | 18 | 2.0 | 6 cats (3%) above 12 mmol/L |
| *Calcium (mmol/L)* | 2.5 | **2.4** | 2.2 | **2.4** | **2.6** | 6.6 | 0.35 | 12 cats (6%) above |
| *Phosphate (mmol/L)* | **1.3** | 1.3 | **0.9** | 1.1 | 1.5 | **3.0** | **0.29** | 20 cats (10%) below and 2 cats (1%) above |
| *Urea (mmol/L)* | 9.3 | **8.7** | 4.7 | **7.5** | **10** | 49.1 | 3.83 | 1 cat below and 50 cats (25%) above |
| *Cholesterol (mmol/L)* | 4.29 | **4.1** | 1.27 | **3.28** | **5.16** | 13.96 | 1.48 | 1 cat below and 57 cats (30%) above |
| *Total Protein (g/L)* | **75.8** | 75.1 | **60.4** | 71.0 | 80.0 | **101.6** | **6.68** | 18 cats (10%) above |
| *Albumin (g/L)* | **30.7** | 30.0 | **23.8** | 29.0 | 32.0 | **43.0** | **3.08** | 1 cat above |
| *Globulin (g/L)* | **45.1** | 45.0 | **40.6** | 28.2 | 49.0 | **77.8** | **7.17** | 30 cats (16%) above |
| *ALKP (U/L)* | 36 | **29** | 9 | **24** | **40** | 219 | 25.5 | 6 cats (3%) below and 10 (5%) above |
| *ALT (U/L)* | 61 | **50** | 10 | **37** | **66** | 514 | 51.4 | 2 cats (1%) below and 28 cats (15%) above |
| *Total bilirubin (µmol/L)* | 3.1 | **3.0** | 0.0 | **0.5** | **4.2** | 17.0 | 2.54 | 1 above |
| *Creatinine (µmol/L)* | 143 | **137** | 41 | **115** | **159** | 735 | 58.0 | 1 cat below, 30 (16%) cats above |
| Reference ranges are not given as the different machines had difference reference ranges instead it has been assessed how many cats fell outside their respective references ranges. Min = minimum values, Q1 = 1^st^ quartile, Q3 = 3^rd^ quartile, Max=maximum value, SD = standard deviation. Bold values indicate which data were parametrically or non-parametrically distributed. | | | | | | | | |

**Table 15:** Summary of routine haematology parameters from 172 middle-aged cats enrolled between February 2017 and October 2019.

| *Red blood cells (RBC)* | *Number of cats* | *Mean ±SD* | *Range* | *Number outside reference ranges* |
| --- | --- | --- | --- | --- |
| RBC count (10^12^/L) | 172 | 8.3 ±1.14 | 4.48-12.49 | 2 (1%) cats below, 3 (2%) cats above |
| Haemoglobin (g/dl) | 172 | 11.9 ±1.65 | 6.3-16.5 | 4 (2%) cats below, 1 above |
| Haematocrit (L/L) | 172 | 0.4 ±0.05 | 0.18-0.67 | 3 (2%) cats below, 4 (2%) cats above |
| Mean corpuscular volume (fL) | 172 | 44.3 ±3.85 | 31.8-53.8 | 3 (2%) below, 17 (10%) above |
| Mean corpuscular haemoglobin concentration (g/dl) | 172 | 32.4 ±1.70 | 21.9-36.6 | 8 (5%) below, 1 above |
| *White blood cells (WBC)* | *Number of cats* | *Median* | *IQR* | *Number outside reference ranges* |
| WBC count (10^9^/L) | 172 | 7.7 | 5.65-9.64 | 36 (21%) cats below, 6 (3%) cats above |
| Neutrophils (10^9^/L) | 165 | 5.3 | 4.05-7.01 | 4 (2%) below, 7 (4%) above |
| Lymphocytes (10^9^/L) | 165 | 1.21 | 0.62-2.25 | 101 (61%) cats below, 1 cat above |
| Monocytes (10^9^/L) | 165 | 0.13 | 0.07-0.21 | 7 (4%) cats above |
| Eosinophils (10^9^/L) | 165 | 0.55 | 0.35-0.73 | 10 (6%) cats above |
| Basophils (10^9^/L) | 165 | 0.02 | 0.01-0.05 | 3 (2%) above |
| Platelets (10^9^/L) | 168 | 247 | 185-330 | 21 (12%) cats had platelet clumping recorded |
| SD= Standard deviation, IQR = interquartile range | | | | |
