# Supplementary material for: Aging in Cats: Owner Observations and Clinical Finding in 206 Mature Cats at Enrolment to the Cat Prospective Aging and Welfare Study
Source: Front Vet Sci. 2022 Apr 4;9:859041. doi: 10.3389/fvets.2022.859041 (PMC9014291; doi:10.3389/fvets.2022.859041)
Supplement: Supplementary file 2 [file Data_Sheet_1.PDF]

Office use only
Owner ID Number:__________________ Feline ID Number:_____________
Annual Visit number: 0 Date:________________________

Enrolment Questionnaire for

Cat PAWS

Prospective Ageing and Welfare Study

Thank you for taking part in Cat P.A.W.S and enrolling your cat in the Feline Healthy Ageing Clinic. Any information you give us will be confidential and used only for the purpose of this study. This study has been approved by the University of Liverpool Ethical Review Committee. You do not have to answer any questions you do not wish to and can withdraw from the study at any time

Instructions:

This Questionnaire is to be completed once for each cat enrolling at the Feline Healthy Ageing Clinic. We ask that it is completed by the person who spends the most time with the cat and cares for them, this person is referred to later as the caregiver. Please complete this questionnaire and bring it with you to your first wellness clinic appointment. If you are unsure of any questions there will be time to discuss them at the appointment. When the option other is given please add in anything that might apply.

Cat's Details

Name: ______________________________ **Date of Birth:**_________________

Age:______________ Please indicate is age is **Known** ☐ or **Estimated** ☐

Sex: **Male** ☐ **Female** ☐ **Unknown** ☐

1. Is your cat neutered?

☐ Yes ***Please answer 1a*** ☐ No ☐ Unknown

a. What age was your cat neutered?

☐ Don't know ☐ 16 – 23 weeks

☐ 7 – 15 weeks ☐ Over 24 weeks (6 months)

2. What colour is your cat?_______________________________________

3. What breed is your cat? ***Domestic refers to a non-pedigree cat or moggie***

☐ Shorted haired domestic ☐ Medium haired domestic

☐ Long haired domestic ☐ Pedigree cat

Please name breed if Pedigree ______________________________________

4. Where did you get your cat from?

☐ Born at home

☐ Pet shop

☐ From pedigree breeder

☐ Rescue Organisation

☐ From non-pedigree breeder / friend / relative

☐ Stray / feral who turned up at house

☐ Other please specify : ___________
____

5. How old was your cat when you obtained them? __________________________

UNIVERSITY OF LIVERPOOL

Please tell us about your cat's lifestyle

6. Which best describes the type of home the cat lives in?

☐ Apartment or Flat ☐ Semi-detached house ☐ Detached house
☐ Terraced House ☐ Other:__________

7. How large is the house? Please give number of bedrooms: __________

8. Does the cat have access to the whole house?

☐ Yes ☐ No

If No please describe where the cat has access to: ________________________
____

9. Is there any outside space?

☐ No ☐ Yes; Yard ☐ Yes; Garden ☐ Yes; Terrace

10. Does the cat currently have access to outside space?

☐ Yes ***answer a-c and proceed to question 11 when finished*** ☐ No ***If No go to question 11***

a. How does your cat access the outside?

☐ Let out when people are home ☐ Let outside when people are out
☐ Free access through cat door / flap or window ☐ Other; Please describe:_____
____

b. How much time does the cat spend outside?

☐ 0% has access but chooses not to go outside
☐ <25% of the time
☐ 25-50% of the time
☐ 50-75% of the time
☐ >75% of the time
☐ 100% of the time, has no indoor access

c. Has this changed as they have gotten older?

☐ No ☐ Yes spends more time outside ☐ Yes spends more time inside ☐ Seasonal change
☐ Don't know

11. Where does the cat go to the toilet?

☐ Litter tray ***If litter tray only please don't complete question 12***
☐ Outside only ***If outside only please move on the question 12***
☐ Litter tray and outside
☐ Other; Please describe: _____

a. How many litter trays are in the house? ______________________________

b. What kind of litter do you use?

☐ Clay based
☐ Paper based
☐ Wood based
☐ Other:______________________

c. Is it?

☐ Clumping
☐ Non-Clumping

f. How would you describe the majority of their faeces?

☐ Dry and firm
☐ Cow pat like
☐ Other; Please describe:_________ _________________
☐ Moist and firm
☐ Runny / Diarrhoea
☐ Formed but soft
☐ Only urinates in tray

g. Does your cat ever miss the litter tray and pass urine or faeces next to the tray?

☐ No never
☐ Yes occasionally
☐ Yes frequently

If Yes is it urine or faeces that miss the tray? ________________________

12. If your cat toilets outside are there suitable areas in your garden/yard for them to toilet in e.g. garden beds?

☐ Yes ***If yes please move on to question 13***
☐ No
☐ Don't know

a. If No, where do they toilet?

☐ Neighbours garden
☐ Don't know
☐ Communal land
☐ Other:_________________

13. Are people home during a normal week?

☐ Always e.g. retired or work from home
☐ Always variable hours *e.g. shift work*
☐ Away predictable hours *e.g. part-time job*
☐ No *e.g. work regular hours*

14. Where does the cat sleep? ***Please mark all that apply***

- ☐ Our bed(s)
- ☐ Couch
- ☐ Cat bed
- ☐ Dog bed
- ☐ Floor
- ☐ Window stills
- ☐ On top of furniture
- ☐ Under furniture
- ☐ In cupboards
- ☐ Nest in garden
- ☐ On top of garden furniture
- ☐ On knees
- ☐ Trees / fences or other high up areas outside
- ☐ On / By radiator
- ☐ Other: ___________

15. If your cat is scared where in the house will they hide? ***Please mark all that apply***

- ☐ Under bed
- ☐ Under furniture
- ☐ On top of furniture
- ☐ In Cupboard
- ☐ Don't know
- ☐ Other: ___________

16. Do you provide / use any of the following? ***Please mark all that apply***

- ☐ Cat bed
- ☐ Scratching post
- ☐ Cat climbing frame
- ☐ Cat toys
- ☐ Cat pheromones *e.g. Feliaway, plug in / spray*
- ☐ None

17. Where does your cat sharpen their claws?

- ☐ Horizontal scratching post
- ☐ Vertical scratching post
- ☐ Furniture
- ☐ Carpet/floor mats
- ☐ Outside
- ☐ Don't know
- ☐ Other:___________

Please tell us about your cat's diet and feeding

18. Please describe how you feed your cat?

Number of Meals:_______________

Usual times of day fed: ___________

Where in the house are they fed?

____

How is the food given?

- ☐ Bowl
- ☐ Automatic feeder
- ☐ Other: *Please describe*___________________

____

19. What Types of food do you feed? Please tick all that apply and give an indication of the percentage for their total diet on an average day.

- ☐ Commercial dry food (kibble) %________
- ☐ Commercial pouch / canned food %________
- ☐ Prepared Raw Food diet %________
- ☐ Tinned Fish %________
- ☐ Fresh Fish %________
- ☐ Cooked Chicken %________
- ☐ Raw Chicken %________
- ☐ Other Raw Meat %________
- ☐ Other Cooked Meat %________
- ☐ Dairy Products %________
- ☐ Cat Treats %________
- ☐ Other:________________ %________

20. Does your cat always finish all of their food?

☐ Yes straight away ☐ Yes but over time between meals ☐ No ☐ Don't know

21. Do they ask for food even when there is food out for them?

☐ Yes ***answer question 23*** ☐ No ☐ Don't know

a. If yes do you have to remind them where their food is?

☐ Yes ☐ No

22. Have you noticed any of the following changes in your cats eating as they have gotten older? *Tick all that apply*

- ☐ Messier
- ☐ Eat more slowly
- ☐ Dropping food when chewing
- ☐ Grinding Teeth
- ☐ Chewing more on one side of the mouth
- ☐ Sniffing food then walking away
- ☐ Leaving more food behind in the bowl
- ☐ Not eating dry food as much
- ☐ None
- ☐ Asking for food but not eating when it is offered
- ☐ Difficulty in bending head to eat or drink
- ☐ Eating more and asking for food more
- ☐ Other: ________________

Please tell us more about your cats drinking

23. How many water bowls or fountains are available for your cat? __________

a. Are these water bowls inside or outside?

☐ Inside only ☐ Inside and outside ☐ Outside only

b. Are the water bowls near the cat's food?

☐ Yes next to ☐ In the same room but not next to ☐ No in another room(s) ☐ Outside only

c. Do you see your cat drinking from other places?

☐ No ☐ Yes Please describe: _____________

d. How often do you see your cat drinking?

☐ Several times a day ☐ Occasionally ☐ Never
☐ Daily ☐ Rarely

e. Have you noticed your cat drinking more as they have gotten older?

☐ Yes ☐ No ☐ Don't know

Please tell us more about your cat's health

24. How best describes your cat's vaccination status?

☐ Had vaccines as a kitten ☐ Vaccinated every year ☐ Not vaccinated
☐ Had some vaccines as an adult but not recently ☐ Don't know if vaccinated or not

If you have a vaccine card please bring it to the appointment

**For Office use only:**
Vaccination dates:
Brands of Vaccine:
Components of Vaccine

25. Do you use flea and tick treatments on your cat?

☐No ***go to question 26*** ☐Yes

a. How frequently do you use them?

☐Monthly ☐In Summer only ☐As required ☐Other:______

b. How would best describe these treatments?

☐Spot on ☐Spray ☐Tablet ☐Other: ______

c. Where do you predominantly buy them?

☐Supermarket ☐Pet Store ☐Vets

d. **When did you last treat your cat?** ____________________________

26. Do you worm your cat?

☐No ***go to question 27*** ☐Yes

a. How frequently do you worm your cat?

☐Every 3 months ☐Yearly ☐Other:___________

☐Every 6 months ☐When required

b. Where do you predominantly buy them?

☐Supermarket ☐Pet store ☐Vets

c. **When did you last treat your cat?** ______________________

27. Do you give your cat any dietary supplements or complementary medications? *Examples: vitamins, joint supplements, herbal products*

☐No ☐Yes ***please list***

____

28. Do you use at any other vet clinics with your cat?

☐No ***go to question 29*** ☐Yes Details: _______________

____

☐Please tick if you would be happy for us to contact them for clinical history

29. Has your cat ever had an episode of ill health?

☐ No ***go to question 30*** ☐ Don't know ***go to question 31***

☐ Yes; ***please give details in the box below and indicate if the cat required a visit to the vet and what treatment was given.***

30. Has your cat required or been recommended dental treatment by the vets ever?

☐ No ***go to question 31*** ☐ Yes

a. What best describes the dental treatment required / advised?

☐ Dental diet ☐ Dental work under anaesthetic ☐ Dental gels ☐ Other: ______

☐ Tooth Brushing ☐ Supplement in water ______

b. Did you go ahead with this recommendation?

☐ Yes ☐ No

c. If dental work was performed were teeth removed?

☐ Yes ☐ Don't know

☐ No ☐ Did not have dental work performed

31. Has your cat ever been tested for any of the following diseases?

Feline Aids (FIV), Feline Leukaemia (FeLV), Feline Herpes Virus or Feline Calivirus

☐ Yes ☐ No ☐ Don't' Know

If yes please indicate what virus(es) they were tested for and if they were positive or not: ____

____

32. Have you noticed your cat vomiting in the last year?

☐ No ***go to question 33*** ☐ Yes ☐ Don't know which cat

a. How often do you see them vomiting or find vomit?

☐ <monthly ☐ Weekly

☐ A few times a month ☐ Several times a week

b. Are hairballs ever seen in the vomit?

☐ Always ☐ Sometimes ☐ Never

33. Have you noticed any of the following physical changes in your cat? ***Check all that apply***

- ☐ Clouding to the eye
- ☐ Coat is not as shiny
- ☐ More matted hair
- ☐ Eyes look more sunken
- ☐ More white hairs
- ☐ Darker flecks in the iris of the eye *(the iris is the coloured part of eye)*
- ☐ Skin lump (s)
- ☐ Thickened nails
- ☐ Feels more bony
- ☐ Breath smells
- ☐ Alterations in the look of the iris
- ☐ None
- ☐ Other: ___________

At what age did you notice these changes? ____________________

34. Have you noticed any of the following activity changes in your cat as they have gotten older? ***Check all that apply***

- ☐ Drinking more water
- ☐ More urine in litter tray
- ☐ Sleeping in different place
- ☐ Going outside less
- ☐ Eating more
- ☐ Eating less
- ☐ Toileting accidents
- ☐ Sleeping more
- ☐ No longer climbing on high furniture
- ☐ Wobbly on legs
- ☐ Jumping up less
- ☐ Appears more stiff when walking
- ☐ Don't like being stroked as much
- ☐ Grooming more
- ☐ Grooming less
- ☐ Difficulty in getting comfortable when resting
- ☐ Difficulty in bending head to eat / drink
- ☐ None
- ☐ Other: ___________ ___________

At what age did you notice these changes?_________________________

35. What body condition do you consider your cat to be in?

- ☐ Thin
- ☐ Lean / skinny
- ☐ Normal
- ☐ Plump / chubby
- ☐ Fat

a. Do you think your cat's body condition has changed as they have gotten older?

- ☐ Yes they are thinner
- ☐ Yes they are fatter
- ☐ No change
- ☐ Don't know

36. What age do you think it is normal for cat's to live to?

- ☐ <10y
- ☐ 10-14yrs
- ☐ 15-20yrs
- ☐ >20yrs

37. What age do you expect your cat to live to?_______________________

UNIVERSITY OF LIVERPOOL

Please tell us more about your cat's behaviour

38. What time of day is your cat most active?

☐ Dawn ☐ Dusk ☐ Don't know
☐ Daytime ☐ Night ☐ Not really active at all

39. How many other cats live in the house? ☐
If none go to question 40

a. Are the cats related to each other?

☐ No
☐ Yes; Explain how: ________________________________

b. Do you see any of the following behaviours between the cats? ***Check all that apply***

☐ Grooming each other
☐ Sleeping together in contact
☐ Rubbing against each other
☐ Sleep in the same area but not in contact
☐ Walk around each other to avoid contact
☐ None
☐ Hissy/growling
☐ Physical aggression
☐ Seldom in the same room at the same time

40. Are there other pets in house? ***Check all that apply***

☐ No ***go to question 41***
☐ Dog(s)
☐ Rabbit(s)
☐ Ferret(s)
☐ Bird (s)
☐ Reptile(s)
☐ Other: ________
____

a. Do you see any of the following behaviours between the cat and the other pets? ***Check all that apply***

☐ Grooming each other
☐ Sleeping together in contact
☐ Rubbing against each other
☐ Sleep in the same area but not in contact
☐ Walk around each other to avoid contact
☐ None
☐ Hissy/growling
☐ Physical aggression/Fighting
☐ Seldom in the same room at the same time

If checked yes to any please list what pets the cat interacts with:______________

41. Does your cat regularly have contact with unfamiliar people?

☐Very rarely ☐Occasionally ☐Very commonly

☐Rarely ☐Commonly

a. Is your cat friendly to new people?

☐ Yes ☐ No

b. Has this changed as the cat has gotten older?

☐ No ☐ Yes; How?________________

42. Does your cat receive any of the following on a regular basis?

☐Food reward training ☐Grooming ☐Nail clipping ☐Tooth brushing

If you Groom or clip your cats nails, has the frequency of this increased as they have aged? ____

43. What things is your cat scared of?

☐Other cat's ☐Washing Machine ☐Thunder ☐Nothing

☐Dogs ☐Children ☐Traffic

☐New people ☐Vacuum cleaner ☐Fireworks ☐Other:_____

44. Have you noticed any of the following changes in your cat's behaviour as they have aged? ***Check all that apply***

☐Waking during the night

☐Meowing more at night

☐Meowing more day and night

☐Becoming grumpy with people

☐Becoming grumpy with other pets

☐More affectionate with people

☐More affectionate with other pets

☐Toileting accidents

☐Appears forgetful

☐Appears confused

☐More demanding of attention

☐Aggressive behaviour for no apparent reason

☐Becoming more fearful or frightened

☐None

If yes at what age? _____________________________________

Finally can you please tell us a little about yourself and your household

45: Please tell us about yourself the primary caregiver for the cat

a. How old are you?

- ☐ Under 18
- ☐ 18-24
- ☐ 25-34
- ☐ 35-44
- ☐ 45-54
- ☐ 55-64
- ☐ 65 and over

b. Are you

- ☐ Female
- ☐ Male

c. Have you owned cat's before?

- ☐ Yes
- ☐ Other people in the house have
- ☐ No, this is my first cat

d. Please check what best represents your educational experience.

- ☐ No additional education
- ☐ GCSE/O Level
- ☐ A Level
- ☐ Undergraduate/vocational
- ☐ Post-graduate / Professional

e. Please circle what best describes your employment.

- ☐ Unemployed
- ☐ Maternity Break
- ☐ Student
- ☐ Part-time work
- ☐ Full-time work
- ☐ Retired
- ☐ Other:___ ________

46. Please tell us about the household

a. Which best describes who lives in the house?

- ☐ Single person household
- ☐ Shared house
- ☐ Couple
- ☐ Young family (children 0-10)
- ☐ Older family (children 11-18)
- ☐ Adult family

b. How long have you lived at your current address

- ☐ < 1 year
- ☐ > 1 year & < 3 years
- ☐ > three years & < 5 years
- ☐ > 5 years

c. Does anyone in the house smoke or has smoked during the last 7 years?

- ☐ Yes now
- ☐ Yes in last 7 years
- ☐ No

47. How best describes your relationship with your cat?

☐ Best Friend ☐ Pet ☐ Other: ________

☐ Family Member ☐ Pest controller ________________

Please add any additional comments you think may be relevant about the wellness of your cat:

Thank you for completing this Questionnaire

FELINE HEALTHY AGEING CLINIC

In partnership with ROYAL CANIN
